# Effects of Purified Vitexin and Iso-Vitexin from Mung Bean Seed Coat on Antihyperglycemic Activity and Gut Microbiota in Overweight Individuals’ Modulation

**DOI:** 10.3390/nu16173017

**Published:** 2024-09-06

**Authors:** Pornlada Yutharaksanukul, Preuk Tangpromphan, Paiboon Tunsagool, Sudathip Sae-tan, Sunee Nitisinprasert, Surasawadee Somnuk, Massalin Nakphaichit, Nut Pusuntisumpun, Bandhita Wanikorn

**Affiliations:** 1 Department of Biotechnology, Faculty of Agro-Industry, Kasetsart University, Bangkok 10900, Thailand; 2 Department of Chemical Engineering, Faculty of Engineering, Kasetsart University, Bangkok 10900, Thailand; 3 Department of Food Science and Technology, Faculty of Agro-Industry, Kasetsart University, Bangkok 10900, Thailand; 4 Department of Sports and Health Science, Faculty of Sports Science, Kasetsart University, Kamphaeng Saen Campus, Nakhon Pathom 73140, Thailand

**Keywords:** vitexin, iso-vitexin, synergistic effect, antihyperglycemic activity, gut microbiota, overweight

## Abstract

Exceeding a healthy weight significantly elevates the likelihood of developing type 2 diabetes (T2DM). A commercially available singular constituent, available as either purified vitexin or iso-vitexin, has been associated with a decreased risk of T2DM, but its synergistic effect has not been reported yet. Vitexin and iso-vitexin were extracted using an ethanol-based solvent from mung bean seed coat (MBCE) and subsequently purified using preparative liquid chromatography (Prep-LC). Eleven mixture ratios of vitexin and/or iso-vitexin were determined for their antioxidant and antihyperglycemic activities. The 1:1.5 ratio of vitexin to iso-vitexin from MBCE demonstrated the most synergistic effects for enzyme inhibition and glucose uptake in HepG2 cells within an insulin-resistant system, while these ratios exhibited a significantly lower antioxidant capacity than that of each individual component. In a gut model system, the ratio of 1:1.5 (vitexin and iso-vitexin) regulated the gut microbiota composition in overweight individuals by decreasing the growth of *Enterobacteriaceae* and *Enterococcaceae*, while increasing in *Ruminococcaceae* and *Lachnospiraceae*. The application of vitexin/iso-vitexin for 24 h fermentation enhanced a high variety of abundances of 21 genera resulting in five genera of *Parabacteroides*, *Ruminococcus*, *Roseburia*, *Enterocloster*, and *Peptacetobacter*, which belonged to the phylum *Firmicutes*, exhibiting high abundant changes of more than 5%. Only two genera of Proteus and *Butyricicoccus* belonging to *Proteobacteria* and *Firmicutes* decreased. The findings suggest that these phytochemicals interactions could have synergistic effects in regulating glycemia, through changes in antihyperglycemic activity and in the gut microbiota in overweight individuals. This optimal ratio can be utilized by industries to formulate more potent functional ingredients for functional foods and to create nutraceutical supplements aimed at reducing the risk of T2DM in overweight individuals.

## 1. Introduction

Type 2 diabetes mellitus (T2DM) is a growing public health challenge worldwide, associated with severe complications that impact healthcare costs and also reduce productivity and quality of life [1,2]. Being overweight or obese has been proven as the predominant modifiable risk factor for T2DM, with 90% of adults diagnosed with T2DM in the overweight or obese categories [3]. In obese individuals, elevated levels of non-esterified fatty acids (NEFAs), glycerol, hormones, cytokines, proinflammatory agents, and other compounds contribute to insulin resistance, leading to progression toward diabetes due to impaired β-cell function [4]. The intestinal microbiome in overweight individuals may play a significant role in the development of T2DM [5]. The gut microbiota perform vital physiological functions in metabolism, particularly in extracting energy and regulating local or systemic immunity. Alterations in their composition and function contribute to the development of obesity and T2DM [6]. However, health-promoting strategies through lifestyle modifications that slow down the progression of T2DM are beneficial, especially in overweight individuals [7]. Current evidence strongly suggests that diets rich in plant foods are associated with a reduced risk of T2DM [8], with the health benefits of plant food intake related to their contained phenolic compounds [9].

Mung bean (Vigna radiata L.) seed coat (MBC) has low economic value as a by-product in the vermicelli industry and is also used as animal feed [3]. Adding value to agricultural by-products is of both scientific and economic interest. Previous studies showed that MBC contained a high total phenolic content at 33.91 ± 1.06 mg gallic acid equivalent/g dry weight [10], with potential health benefits as a complementary and alternative functional ingredient providing antioxidant, anti-inflammatory, anti-diabetic, and hepatoprotective activities [11]. Over the last decade, several studies have focused on isolating and identifying bioactive compounds in MBC [12,13]. Vitexin and iso-vitexin were identified as the two major phenolic compounds in MBC at 15.28 mg/g and 23.74 mg/g, respectively [14]. Purified vitexin and iso-vitexin, as individual constituents extracted from specific plant sources such as sea bilberry, passionflower, candle millet, and chaste tree, have recently attracted increased attention due to their diverse pharmacological effects [15].

Several studies have indicated that efficacy is higher using a combination of compounds compared to single active constituents [16], with the ratios of the combined compounds shown to significantly influence the extent of synergy [17]. However, most vitexin and iso-vitexin research has focused on studying individual constituents rather than examining their combined effects, and investigations into the effects of constituents from MBC have also been limited [18]. Most dietary phenolics are metabolized by colonic microbiota before absorption, thereby inducing indirect health effects in mammals through the action of these microorganisms [19]. However, studies on the biological properties of human microbiota after MBC phenolic consumption in overweight individuals are limited, while the concept of synergy is of great significance when designing functional foods and nutraceuticals for promoting human well-being and preventing diseases [20].

Therefore, this research investigated the optimal mixture ratio of vitexin and iso-vitexin separated from MBC to maximize their antihyperglycemic activities in an insulin resistance system. The effects of these compounds on gut microbiota in a human gut model were also studied for overweight individuals. The results will provide a more comprehensive understanding of the synergistic, combinatory, or antagonistic effects of MBC phenolics on biological activity to improve insulin resistance and shed light on the relationship between these compounds and the gut microbiome. This understanding will aid in the development of strategies to use by-products for ameliorating T2DM in overweight individuals and promote the usefulness of MBC by-products as valuable functional ingredients in the industrial food sector for the production of functional foods and nutraceuticals.

## 2. Materials and Methods

### 2.1. Chemicals and Reagents

Standard reagents such as vitexin, iso-vitexin, Trolox, alpha-amylase (2 U/mg protein), alpha-glucosidase (14 U/mg protein), acarbose, MTT, and 2-NBDG were purchased from Sigma-Aldrich (St. Louis, MO, USA). Dulbecco’s modified Eagle’s medium (DMEM) was obtained from Hyclone (Logan, UT, USA). Fetal bovine serum, non-essential amino acid cell culture supplement, and Penicillin–Streptomycin were bought from Thermo Fisher Scientific (Bangkok, Thailand). Other chemicals were purchased from Bang Trading 1992 Co., Ltd. (Bangkok, Thailand) and A&A Reagent Ltd. (Songkhla, Thailand).

### 2.2. Plant Material

Mung bean seed coat (MBC) was obtained from Kittitat Co., Ltd. in Bangkok, Thailand, and transported to the Laboratory of the Biotechnology Department, Kasetsart University.

### 2.3. Preparation of Vitexin and Iso-Vitexin from Mung Bean Seed Coat

The extraction method followed that outlined by Jaree et al. [12]. A 150 g aliquot of MBC was dried at 40 °C for 24 h. The dried sample was then subjected to dry grinding, followed by sieving through mesh sizes 0.250 mm and 0.177 mm, and was stored in vacuum-sealed bags at 3–5 °C. One gram of dried sample was then extracted with 23 mL of 55% ethanol at 65 °C under agitation at 1400 rpm for 15 min, and the filtrate was analyzed by HPLC. The chromatogram of the extracted sample was compared with the chromatogram of the standard solution obtained using the appropriate mobile phase and operating conditions of HPLC to confirm that vitexin and iso-vitexin were the major extract solution components.

### 2.4. Quantitative Determination of Phenolic Compounds in Mung Bean Seed Coat Extract (MBCE)

#### 2.4.1. Quantitative Analysis of MBC Phenolic Compounds

Total phenolic compounds were tested by the Folin–Ciocalteu method [21]. Briefly, 20 µL of crude extract solution was mixed with 100 µL of 10% (*w*/*v*) Folin–Ciocalteu reagent. After 7 min, 80 µL of Na_2_CO_3_ (7.5%) was added to the mixture and incubated in the dark at room temperature (25 °C) for 1 h. The absorbance was measured by a microplate reader at 760 nm against a blank without extract, with data expressed as mg/g of gallic acid equivalent to milligrams per gram (mg GAE/g) of dry extract.

#### 2.4.2. Separation of Vitexin and Iso-Vitexin from MBCE

Vitexin and iso-vitexin contained in the liquid extract were isolated using preparative liquid chromatography (Prep-LC) according to the modified method of Jaree et al. [12]. They were separated on a C18 column (4.6 mm × 250 mm, Kromasil, Nouryon, Sweden) using an ethanol/water ratio of 15:85% *v*/*v* as a mobile phase at 40 °C with a flow rate of 1 mL/min and a 330 nm UV detector wavelength. One milliliter of the liquid extract was loaded into the column using the HPLC pump, and the mobile phase was fed to the column to elute all of the compounds. The outlet products were fractionated corresponding to the times of vitexin and iso-vitexin signals observed from the UV detector. The collected vitexin and iso-vitexin fractions were then subjected to an HPLC analysis to determine their concentrations.

#### 2.4.3. HPLC Analysis

The Thermo Dionex Ultimate 3000 high-performance liquid chromatography system (Thermo Fisher Scientific Inc., Waltham, MA, USA) was used, along with a C-18 column (Avantor, 250 × 4.6 mm). The temperature was set at 40 °C with a flow rate of 1 mL/min in 15%v/v aqueous ethanol. The injection volume was 10 µL and was detected using a UV detector at 330 nm. The sample was filtered using a 0.22 µm nylon filter before being injected.

### 2.5. Synergistic Reaction Mixture Preparation

The synergistic activity assay was carried out following the method of Gourineni et al. [22]. Twenty-five milligrams of each bioactive compound and purified compound of MBC extract was dissolved in 100 mL of autoclaved water, and was filtered using Whatman No.1 paper. Combination solutions were prepared using two bioactive agents (vitexin/iso-vitexin) at ratios of 0:1, 1:0, 1:1, 1:1.5, 1:2, 1:2.5, 1:3, 1.5:1, 2:1; 2.5:1, and 3:1 *v*/*v*.

### 2.6. Determination of Antioxidant Activity

#### 2.6.1. The Determination of Antioxidant Activity Using the DPPH Method

The DPPH (1-(2,6-dimethylphenoxy)-2-(3,4-dimethoxyphenylethylamino) propane hydrochloride) assay was measured according to the protocol described by Brand-Williams et al. [23]. One hundred microliters of the sample was mixed with 100 µL of 0.4 mM methanolic solution containing DPPH radicals, and the mixture was incubated in the dark at room temperature for 30 min. Absorbance measurements were conducted at 517 nm. The DPPH radical scavenging activity was calculated using Equation (1).

(1) $$DPPH\;radical\;scavenging\;activity\;\left(\%\right)=[(A_{c}{-A}_{t}/A_{c})]\times 100\%$$

A*_t_*: Absorbance of the test well and A*_c_*: Absorbance of the untreated well (control).

#### 2.6.2. The Determination of Antioxidant Activity Using the ABTS^•+^ Method

The ABTS^•+^ (2,2′-azino-bis(3-ethylbenzothiazoline-6-sulfonic acid) assay was conducted following Re et al.’s method [24] with some modifications. Seven mM ABTS stock solution was mixed with 2.45 mM potassium persulfate (final concentration), and the mixture was allowed to stand in the dark at room temperature for 12 to 16 h before use. The ABTS^•+^ solution was diluted with deionized water and 95% ethanol (1:1) to an absorbance of 0.70 ± 0.02 at 734 nm. Fifty microliters of the sample was mixed with 150 µL of diluted ABTS^•+^ solution and the absorbance was measured after 15 min at 734 nm. The ABTS radical scavenging activity was calculated using Equation (2).

(2) $$ABTS\;radical\;scavenging\;activity\;(\%)=[(A_{c}{-A}_{t}/A_{c})]\times 100\%$$

A*_t_*: Absorbance of the test well and A*_c_*: Absorbance of the untreated well (control).

#### 2.6.3. The Determination of Antioxidant Activity Using the FRAP Method

The FRAP (ferric reducing antioxidant power) assay was modified from Benzie and Strain [25]. Six milliliters of working FRAP reagent (0.1 M acetate buffer:0.02 M FeCl_3_:0.01 M TPTZ = 10:1:1) was prepared daily and mixed with 20 µL of the extract sample. The absorbance at 593 nm was measured after 30 min of incubation at 37 °C and reported as mg Trolox C equivalent per gram of dry weight.

### 2.7. Determination of Antihyperglycemic Activity

#### 2.7.1. Alpha-Amylase Inhibition Assay

The alpha-amylase inhibitory potential was investigated by reacting different concentrations of the extracts with the alpha-amylase enzyme and starch solution [26]. A mixture of 50 µL phosphate buffer (100 mM, pH 6.8) and 20 µL of the samples containing 10 µL of alpha-amylase (2 U/mL) was incubated at 37 °C for 20 min in a 96-well plate. Then, 10 µL of 1% starch solution was added and further incubated for 30 min. Thereafter, 100 µL of dinitrosalicylic acid (DNS) was added, and the mixture was boiled at 95 °C for 15 min. Acarbose (2–10 µg/mL) was used as the positive control (Equation (3)).

#### 2.7.2. Alpha-Glucosidase Inhibition Assay

The alpha-glucosidase inhibitory activity of the sample was conducted following the standard method with slight modifications [27]. First, 50 µL of phosphate buffer (100 mM, pH 6.8), 10 µL of alpha-glucosidase (1 U/mL), and 20 µL of samples and positive control (acarbose) were incubated at 37 °C for 15 min in a 96-well plate. Then, 20 µL of 5 mM substrate (4-nitrophenyl β-D-glucopyranoside) was added and left to incubate for 20 min. The reaction mixture was stopped after incubation by adding 50 µL of 0.1 M sodium carbonate. The release of p-nitrophenol into the reaction mixture relating to the activity of the enzyme was read at a wavelength of 405 nm (Equation (3)).

(3) $$\%\;Inhibition=[(1-(A_{t}/A_{c}))]\times 100\%$$

A*_t_*: Absorbance of the test well and A*_c_*: Absorbance of the untreated well (control).

#### 2.7.3. Determination of Synergistic Activity

The synergistic activity assay was performed according to a modified method of Rumjuankiat et al. [28]. The fractional inhibitory activity (FIA) index (ΣFIAs) was calculated as follows: ΣFIA = FIA-X + FIA-Y, where FIA-X is the enzyme inhibition in the combination/enzyme inhibition activity of vitexin alone and FIA-Y is the enzyme inhibition in the combination/enzyme inhibition activity of iso-vitexin alone. The FIA was interpreted as synergy when it was more than 2.

#### 2.7.4. HepG2 Cell Cultures for Glucose Uptake

HepG2 cells (courtesy of Asst. Prof. Sudathip Sae-tan, sourced from ATCC) were used to determine the effects of compounds on glucose uptake. HepG2 cells were cultured in Dulbecco’s modified Eagle’s medium (DMEM) (1 g/L glucose) and supplemented with 10% fetal bovine serum, 1% non-essential amino acid, and 1% Penicillin–Streptomycin. The cells were incubated at 37 °C in a humidified atmosphere with 5% CO_2_. The medium was changed every 2 days and subcultured once the cells reached 70–90% confluence.

#### 2.7.5. Cytotoxicity Assay

Cell viability was determined by the 3-(4,5-dimethylthiazol-2-yl)-2,5 diphenyltetrazolium bromide (MTT) assay following Choi et al.’s method [29] with some modifications. HepG2 cells were seeded in a 96-well plate at 3 × 10^4^ cells/well and cultured for 24 h. The cell culture medium was removed from each well, and the samples were added to the desired concentration of 100 µL/well and incubated for 24 h before all samples were removed. Subsequently, 100 µL of MTT (0.25 mg/mL) was added to each well and incubated at 37 °C for 45 min. The MTT-containing medium was gently removed and replaced with 100 µL of dimethyl sulfoxide (DMSO) to dissolve the formazan crystals. The absorbance was measured at 570 nm with a microplate reader, and the results were presented as percentages of the control values (Equation (4)).

(4) $$\%\;Cell\;viability=[(A_{t}/A_{c})]\times 100\%$$

A*_t_*: Absorbance of the test well and A*_c_*: Absorbance of the untreated well (control).

#### 2.7.6. Glucose Uptake in IR-HepG2 Cells

The glucose uptake in HepG2 cells was assessed by the 2-deoxy-2-[(7-nitro-2,1,3-benzoxadiazol-4-yl)amino]-D-glucose (2-NBDG) assay, as described by Zou et al. [30]. HepG2 cells were cultured in a 96-well plate at 3 × 10^4^ cells/well for 24 h. The medium was changed and replaced with 100 µL of serum-free medium containing 1 µM insulin for 24 h to induce insulin resistance. After that, all the cell culture medium was removed from each well, replaced with samples, and it was incubated for another 24 h. All samples were removed and replaced with 100 nM insulin in a serum-free medium and incubated for 30 min. Then, 100 µL of 2-NBDG (40 µM in serum-free medium) was added and incubated for another 30 min. The cells were cleaned with chilled phosphate-buffered saline for 3 cycles before a fluorescence analysis at wavelengths of 485 and 528 nm (Equation (5)).

(5) $$\%\;Glucose\;uptake=[(A_{t}/A_{c})]\times 100\%$$

A*_t_*: Absorbance of the test well and A*_c_*: Absorbance of the untreated well (control).

### 2.8. Fecal Sample Collection

This study was approved by the Ethics Committee of Kasetsart University (license number COA64/068) and registered with the Thai Clinical Trials Registry (TCTR20220204007). Fresh fecal samples were collected from three healthy overweight male Thai donors. The overweight donors met the following criteria: individuals aged 25–60 years with a Body Mass Index (BMI) ranging from 23 to 30 kg/m^2^ who had not taken antibiotics, those who had not used probiotic and prebiotic products in daily life for at least for three months, and those who had no gastrointestinal disorders. Written informed consent was obtained from all the donors before sample collection. The fresh fecal samples were transported under anaerobic conditions and used within 1 h. Five grams of the fecal sample was mixed with 4 vol of sterile phosphate-buffered saline (PBS). A fecal solution was transferred into the batch fermentation, which was used as the human gut model.

### 2.9. Simulation of Human Gut Model

Batch culture fermentations (100 mL each) were set up using fecal slurries from the three Thai donors. A basal medium of 70 mL was prepared following the method of Onumpai et al. [31]. The medium was sterilized at 120 °C for 20 min before being aseptically added into sterile fermenters, which were maintained at a pH of 6.65 to 6.95 and 37 °C under an anaerobic atmosphere by continuously feeding N2 gas. The fecal slurry was incubated in the fermenters for 1% (*w*/*v*) of the total working volume. The optimal ratio of vitexin and iso-vitexin at 0.1% (*w*/*v*) in the fermentation media was used in each test, and the fecal solution with basal medium was used as the control. All contents from the human gut model at 24 h of fermentation were collected as samples and kept at −80 °C.

### 2.10. DNA Extraction, 16s rRNA Gene Sequencing, and Analysis

The microbial DNA extraction method followed Pusuntisumpun et al.’s method [32]. The quality and quantity of microbial DNA were measured using a NanoDrop™2000/2000c spectrophotometer (ThermoFisher Scientific, Waltham, MA, USA). Samples were vortexed and the contents were spun down before their nucleic measurement. A blank was set at 2 µL of elution buffer and all the bacterial DNA were kept at −20 °C.

The metagenomic analysis was carried out with a minor modification of the manufacturer’s methods according to Pusuntisumpun et al. [32]. Bacterial DNA amplification from the V3-V4 region of 16s rDNA was conducted via a polymerase chain reaction (PCR) using forward primer 5′-CCT ACG GGN GGC WGC-3′ and reverse primer 5′-GA CTA CHV GGG TAT CTA ATC C-3′. The sequences of the 16s rRNA gene were trimmed to the low-quality bases and adapters to create clean sequences. Usearch version 11.0.667 (available at https://drive5.com/usearch/ accessed on 15 February 2024) was used to analyze the filtered sequences from all samples, identify operational taxonomic units (OTUs), and determine microbial compositions.

### 2.11. Statistical Analysis

Antioxidant and antihyperglycemic activities were performed using IBM SPSS Statistics for Windows Version 23. Significance was analyzed by one-way ANOVA, with data expressed as mean ± SEM, *n* > 3. Metagenomics analyses were performed using XLSTAT version 2019.2.2. The change in gut microbiota at 24 h was compared using the parametric Student’s *t*-test for multiple sample comparisons. Statistical significance was defined as *p*-values < 0.05.

## 3. Results

### 3.1. Quantitative and Qualitative Characters of Phenolic Compounds in Mung Bean Seed Coat Extract (MBCE)

**Figure 1:**
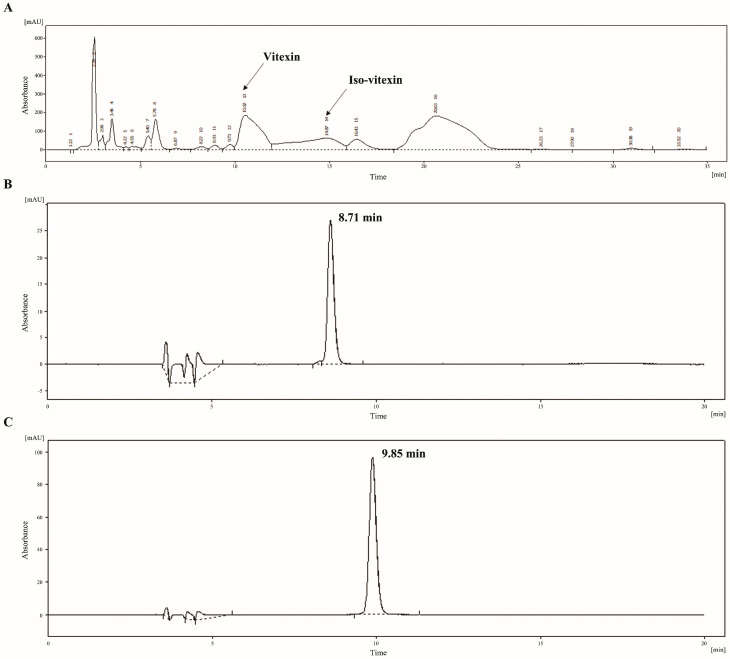
Chromatogram of (**A**) crude extract, (**B**) vitexin fraction (only vitexin peak was observed), and (**C**) iso-vitexin fraction (only iso-vitexin peak was observed) from MBCE.

The MBCE weight was 0.4410 g, with yields of 11.03% after ethanol extraction. The MBC contained a high total phenolic content ranging from 39.21 ± 0.17 to 44.04 ± 0.08 mg gallic acid equivalent/g dry weight. Vitexin and iso-vitexin peaks from MBCE are shown in the Prep-LC chromatogram (Figure 1A). The retention time of vitexin and iso-vitexin peaks in the MBCE recorded at 330 nm were 10.52 and 14.87 min, respectively. To confirm vitexin and iso-vitexin fractions by an HPLC analysis, the retention times of vitexin and iso-vitexin from the collected fractions were 8.71 and 9.85 min (Figure 1B,C). The concentrations of vitexin and iso-vitexin were 144 and 529 µg/mL, respectively.

### 3.2. Antioxidant Activity

The results in Table 1 show the IC50 values of phenolic compounds at different vitexin/iso-vitexin ratios for DPPH^•^ radical and ABTS^•+^ radical scavenging activities and the FRAP assay. A lower IC50 value indicates higher antioxidant capacity. The ratios 0:1 and 1:0 (vitexin/iso-vitexin) showed significantly lower IC50 values of DPPH^•^ radical and ABTS^•+^ radical scavenging activities compared to the other ratios. The 1:1 ratio gave the highest IC50 value of inhibiting DPPH^•^ radical and ABTS^•+^ radical. The ferric ion reducing antioxidant potential (FRAP) of crude MBCE and eleven different ratios of vitexin and iso-vitexin from MBCE were also estimated for their ability to reduce TPTZ-Fe (III) to TPTZ-Fe (II). A significantly higher total antioxidant capacity was observed in the ratios of vitexin/iso-vitexin 0:1, 1:0, and 1:1 (41.04 ± 0.04, 37.81 ± 0.04, and 39.06 ± 0.03 mg Trolox/g MBC, respectively).

### 3.3. Enzyme Inhibitory Activity and Synergistic Activity of Vitexin and Iso-Vitexin

The inhibitory effects of different ratios of vitexin and iso-vitexin from MBCE, as well as the commercial standard of vitexin/iso-vitexin, against alpha-amylase and alpha-glucosidase were explored. The alpha-amylase inhibition assay showed that the ratio of 1:0 (vitexin/iso-vitexin) of MBCE and the commercial standard gave the highest inhibition at 71.31% and 70.84% of enzyme activity, respectively, at 31.93 µg/mL (73.85 mM). Compared with acarbose (60 µg/mL; 93.02 mM), the percentage inhibitory activity was not significantly different except the combination treatment 1:2.5 (Table 2). However, various synergistic activities occurred at the combination of vitexin and iso-vitexin. The results show that the combination treatments of vitexin and iso-vitexin from MBCE at 1:2, 1.5:1, 2:1, and 3:1 exerted the highest FIA (Functional Inhibitory Activity) index of more than 2.4 times, while the ones at 1:1.5 and 2:1 did so for more than two times. Interestingly, no synergy occurred in the combination of commercial standards.

Considering alpha-glucosidase, all combination treatments of MBCE and the commercial standard exhibited similar inhibition activities, except the treatment of 1:2 and 3:1 of MBCE, which had significantly higher activities as shown in Table 3. However, the ratios of 1:0 and 1:1.5 (vitexin/iso-vitexin) of MBCE and the commercial standard showed potential high activities to inhibit alpha-glucosidase compared to acarbose. By contrast, the 1:1 and 2:1 ratios of MBCE and the commercial standard showed the lowest potential of inhibiting alpha-glucosidase. The combination of vitexin and iso-vitexin had no synergy except the combination treatments of 1:2 and 3:1 from the commercial standard. When the inhibition activities of both enzymes were taken into account, the combination of vitexin/iso-vitexin at the 1:0, 0:1, and 1:1.5 ratios were considered for further studies.

### 3.4. Cytotoxicity of HepG2 Cells

**Figure 2:**
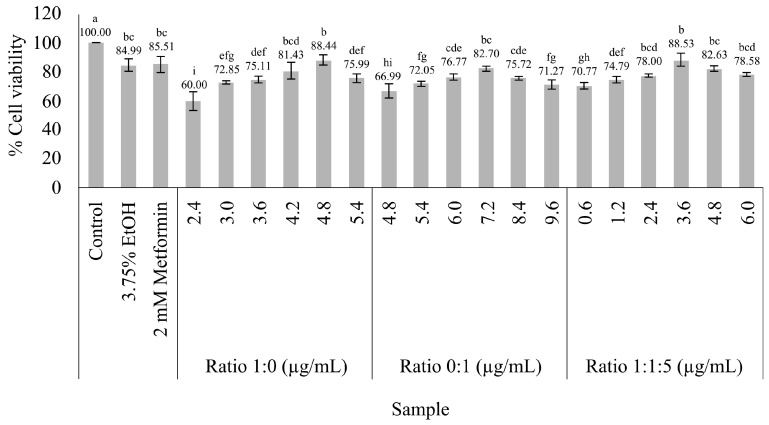
Cytotoxicity of different vitexin/iso-vitexin ratios on the viability of HepG2 cells. The results are the mean ± standard deviation of three measurements. Different letters indicate significant differences within a group (*p* < 0.05).

The cytotoxicity results of vitexin and iso-vitexin ratios towards HepG2 cells after 24 h of treatment indicated that at ratios of 0:1, 1:0, and 1:1.5, % cell viability was above 80% at concentrations below 7.2 µg/mL (Figure 2).

### 3.5. Glucose Uptake in HepG2 Cell

**Figure 3:**
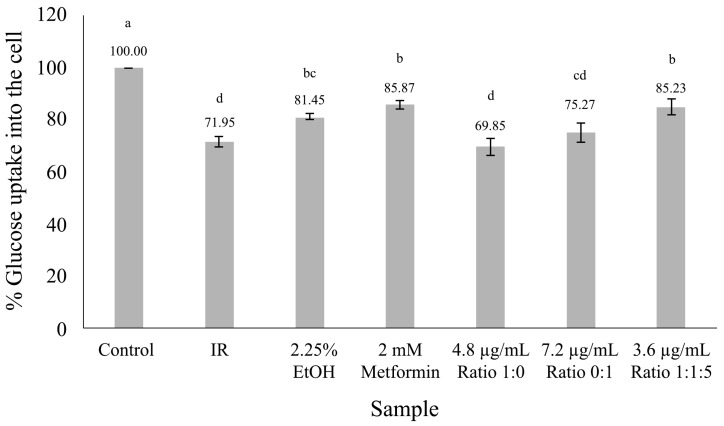
Glucose uptake in HepG2 cells. The results are the mean ± standard deviation of three measurements. Different letters indicate significant differences within a group (*p* < 0.05). Control refers to normal cells and IR refers to insulin-resistant cells.

Figure 3 demonstrates that in an insulin-resistant model, the 1:1.5 ratio of vitexin to iso-vitexin from MBCE markedly enhanced the glucose uptake into HepG2 cells compared to individual compounds. This effect showed a similar efficacy to metformin, with no statistically significant difference observed (*p* > 0.05).

### 3.6. Impact of Vitexin/Iso-Vitexin in Gut Microbiota

**Figure 4:**
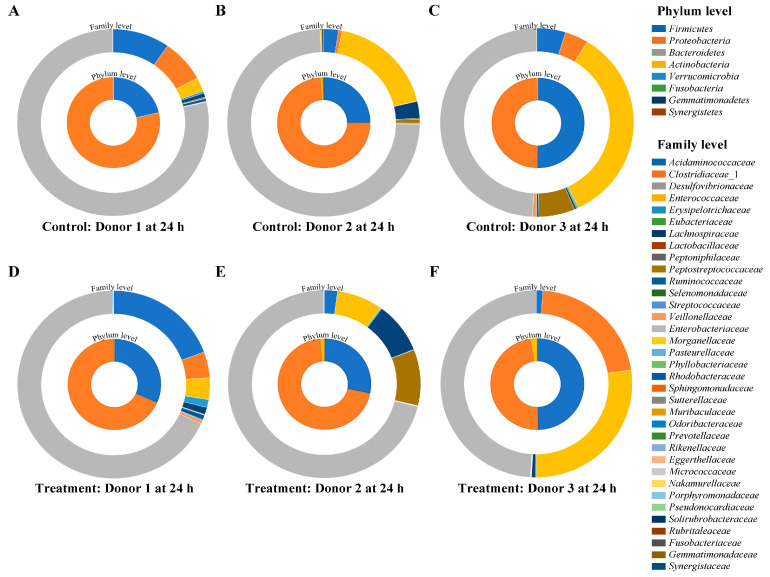
Relative abundance of phylum and family levels at 24 h, where (**A**–**C**) represents the control group and (**D**–**F**) represents the treatment group.

**Figure 5:**
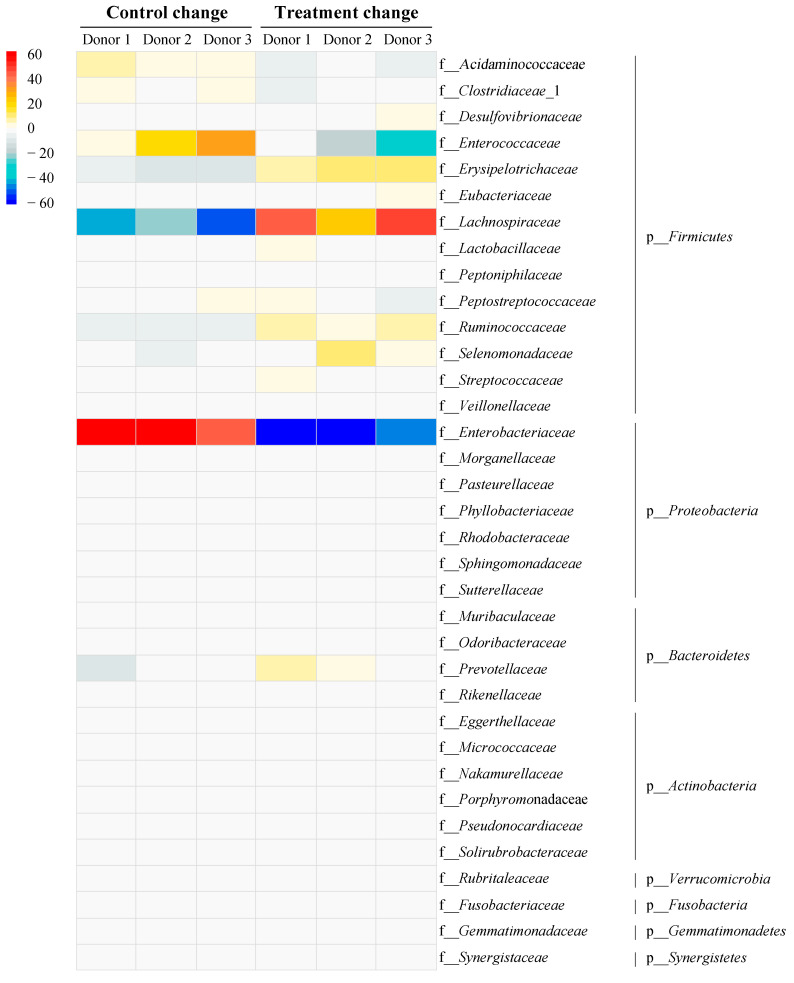
Relative abundance changes at family levels.

Vitexin and iso-vitexin from MBCE at a ratio of 1:1.5 were studied in a simulated human gut model. Figure 4 shows the phylum and family-level composition of gut microbiota between the three donors at 24 h. Phylum *Firmicutes* and *Proteobacteria* showed the highest abundance in the control group across all donors. After treatment with 1:1.5 of vitexin/iso-vitexin, there were no differences at the phylum level compared to the control group. At the family level, *Enterobacteriaceae* abundance decreased in donors 1 and 2, while it remained similar to the control group in donor 3. In addition, changes were observed at the family level by a heat map analysis (Figure 5). The control group showed an increase in the abundance of *Enterobacteriaceae* and *Enterococcaceae*. After treatment with 1:1.5 of vitexin/iso-vitexin, an increase in *Ruminococcaceae*, as well as *Lachnospiraceae*, and a suppression of *Enterobacteriaceae* and *Enterococcaceae* were observed. A total of 117 genera were identified (Supplementary Table S3) and showed a similar pattern across each treatment, but the percentage change after 24 h of fermentation in the gut model differed. Twenty-one genus increases were obtained by vitexin/iso-vitexin supplementation (Table 4). Only five genera of *Parabacteroides*, *Ruminococcus*, *Roseburia*, *Enterocloster*, and *Peptacetobacter*, which belonged to the phylum *Firmicutes*, exhibited high abundant changes of more than 5%, while the ones of *Proteus* and *Butyricicoccus*, belonging to *Proteobacteria* and *Firmicutes*, decreased. This implied that the application of vitexin/iso-vitexin enhanced the number of higher abundances, resulting in more microbial diversity.

## 4. Discussion

Obesity-induced insulin resistance and disrupted glucose metabolism contribute to the dysregulation of cellular carbohydrate and lipid metabolism, leading to the development of diabetes [33]. Vitexin and iso-vitexin have recently received increased attention due to their wide-ranging pharmacological effects including antioxidant, anti-diabetic, anticancer, anti-inflammatory, antihyperalgesic, and neuroprotective properties [18], but most studies have only investigated single constituents. Several studies have indicated that disease prevention is more likely to be achieved through the utilization of a combination of compounds, rather than relying solely on single active constituents [17]. The ratios of the combined compounds significantly affected the magnitude of the synergy [16]. A possible mechanism involved in these pharmacological effects could be the modulation of gut microbiota in a synergistic way. However, the characterization of the gut microbiota in overweight individuals remains insufficiently established, and some results are inconsistent [34,35]. The concept of synergy is of great significance when designing functional foods and nutraceuticals for promoting human well-being and preventing diseases.

Mung bean seed coat (MBC), a by-product of the vermicelli industry, is rich in vitexin and iso-vitexin as its two major phenolic compounds [14]. Our results confirmed this, suggesting that MBC may serve as a promising source for obtaining purified vitexin with a high yield. The amount of purified phenolic compounds depends on the extraction and purification method [36]. This study employed ethanolic extraction coupled with a Prep-LC system for the purification step.

Free radicals generated within the body are partially implicated in the etiology of T2DM, as oxidative stress disrupts insulin-mediated intracellular signaling pathways, ultimately leading to insulin resistance in overweight individuals [37]. An adequate intake of antioxidants plays a crucial role in safeguarding against the disease [38]. Phenolic compounds are important antioxidants that exhibit activity by several mechanisms such as donating hydrogen atoms to free radicals, scavenging other reactive species, or binding transition metal ions (especially iron and copper) [39]. Our results demonstrated that vitexin and iso-vitexin exhibited antioxidant activity. Their radical scavenging activity was attributed to the phenolic hydroxyl group in the 4′ position on the B-ring [40]. However, combinations of vitexin and iso-vitexin did not provide synergistically enhanced antioxidant activity regarding their self-inhibition [41].

Postprandial hyperglycemia can be delayed by reducing glucose absorption through the inhibition of carbohydrate-hydrolyzing enzymes in the gastrointestinal tract [42,43]. Alpha-amylase catalyzes the cleavage of alpha-(1-4) glycosidic binding to dextrin, maltose, or maltotriose, while alpha-glucosidase catalyzes the hydrolysis of 1–4 linked alpha-glucose and generates glucose molecules [44]. Many studies have searched for more effective and safe inhibitors of alpha-glucosidase and alpha-amylase from natural sources to develop nutraceuticals that can prevent and manage diabetes [45]. One study demonstrated that vitexin displayed strong inhibition against alpha-glucosidase [46], which aligned with our findings. In synergistic activity, it was found that an increase or decrease in the ratio between vitexin and iso-vitexin affects the FIA value, which represents the synergistic or antagonistic activity of enzyme inhibitions. Cao et al. [47] showed that the glycosylation of flavone can modify the distribution and density of the electron cloud among the rings and introduce steric hindrance, which weakens the binding of flavone to the enzyme, leading to a reduction in its inhibitory activity. The glycosylation of flavonoids reduces their inhibitory effect on α-amylase and α-glucosidase, with the extent of reduction depending on the position of the glycosylation and the class of sugar moiety [48].

Insulin resistance in obesity is characterized by reduced insulin-triggered glucose transport and processing in both fat cells and skeletal muscle, along with ineffective regulation of glucose production in the liver [49]. Polyphenols also impact peripheral glucose absorbed in insulin and non-insulin-sensitive tissues [50]. Insulin typically facilitates anabolic metabolic processes in the liver by promoting glucose uptake and lipid synthesis. Overweight individuals with insulin resistance are incapable of adequately suppressing hepatic glucose production, thereby resulting in hyperglycemia [51]. Prabhakar and Doble [52] showed that phenolic acids excite glucose uptake with a similar absorption method to metformin, while phenolic compounds increase the catalytic activity of glucose phosphorylation and also influence the function of glucose and insulin receptors by increasing the expression of GLUT2, a glucose transporter, in pancreatic β (insulin-producing) cells [53,54,55]. Moreover, Pavasutti et al. [11] demonstrated that mung bean seed coat water extract (MSWE) showed the potential to restore insulin resistance in insulin-resistant HepG2 cells, as demonstrated by the enhanced uptake of glucose into the cells. The MSWE consisted of vitexin and iso-vitexin. These results demonstrated that mixtures of vitexin and iso-vitexin in our study of MBCE showed greater potential for glucose uptake into HepG2 cells.

The maximum dissolved concentration of ethanol in the ratios showed the absence of significant cytotoxicity, while the percentage of cell viability was significantly inhibited in samples at higher concentrations of ethanol compared with the control group [56]. Furthermore, ethanol impacts the uptake of glucose by increasing peripheral insulin resistance [57,58] and reducing GLUT4 expression, thus lowering insulin sensitivity and glucose tolerance [59].

The human microbiome comprises a diverse ecosystem of commensal, symbiotic, and potentially pathogenic microorganisms that cohabit with our body environment [60]. The primary gut microbiota predominantly falls within four major phylum (phyla): Firmicutes, *Bacteroidetes*, *Proteobacteria*, and *Actinobacteria* [61,62]. Relman and Falkow [63], Jandhyala et al. [64], Hasan and Yang [65], and Somnuk et al. [66] reported that age, diet, antibiotics, probiotics, and prebiotics significantly impacted microbiome diversity. All donors in this study had a similar Body Mass Index (BMI) and health status, as per the inclusion criteria, but variations were observed. The fecal gut microbiota exhibited distinct patterns among the three volunteers, possibly attributed to host genetics, lifestyle choices, and dietary habits. Under typical physiological conditions, the gut microbiota is predominantly composed of *Firmicutes* (64%), followed by *Bacteroidetes* (23%), *Proteobacteria* (8%), and *Actinobacteria* (3%). By contrast, the predominant phylum encountered in obesity and T2DM was *Proteobacteria* [67,68], which aligned with our findings.

Previous studies found that *Enterobacteriaceae* are typically present in low abundances in healthy humans, constituting just 0.1–1% of the relative abundance on average [69], while *Enterococcaceae* typically comprise less than 1% of adult microflora [70]. Karlsson et al. [71] reported significantly higher concentrations of *Enterobacteriaceae* in obese or overweight humans compared to those who were not overweight, while Rodriguez et al. [72] also noted a similar trend in *Enterococcaceae*. A significant increase in *Enterococcaceae* and *Enterobacteriaceae* was observed in a patient with T2DM [73,74], indicating a correlation between BMI, T2DM, and a high abundance of *Enterobacteriaceae* and *Enterococcaceae*. Biddle et al. [75] and Andrade et al. [76] reported that the *Firmicutes* phylum, the *Lachnospiraceae* and *Ruminococcaceae* hydrolyze starch and other sugars are used to produce butyrate and short-chain fatty acids (SFCAs), which also benefit hosts in preventing diabetes. Previous studies showed that cranberry extract, which is rich in polyphenols, reduced the abundance of *Enterobacteriaceae* in a human gut model [77], while apigenin effectively inhibited *Enterococcaceae* [78]. *Enterobacteriaceae* and *Enterococcaceae* play a key role in the onset and development of diabetes [79]. Additionally, Larsen et al. [80] reported that increased levels of the Parabacteroides genus are linked to enhanced insulin sensitivity, probably contributing to the regulation of blood sugar levels by improving the body’s insulin response. These previous studies support our findings, including the beneficial effect of butyric acid in promoting high-abundance genera such as *Parabacteroides*, *Ruminococcus*, *Roseburia*, *Enterocloster*, and *Peptacetobacter*. Aguirre et al. [81] studied gut microbiota from lean and obese individuals, and proposed that a high abundance of *Roseburia* genera may have a potential role in obesity reduction potential correlating to diabetic symptoms. These results demonstrated that the ratio of vitexin and iso-vitexin at 1:1.5 in MBCE showed the potential to improve levels of beneficial bacteria, while inhibiting pathogenic bacteria.

## 5. Conclusions

This is the first study to demonstrate the synergistic effects of individual vitexin and iso-vitexin constituents separated from MBC ethanol-based extract on antihyperglycemic activities in an insulin-resistant system and gut composition in overweight individuals. Our results demonstrated that the combination of purified vitexin and iso-vitexin exhibited superior antihyperglycemic potential compared to their individual counterparts. This beneficial effect of phenolic compounds may be exerted through their interaction with the gut composition. This comprehension will facilitate the formulation of tactics to mitigate T2DM in overweight individuals and advocate for the utility of MBC by-products as valuable functional ingredients within the industrial food sector, specifically for the production of functional foods and nutraceuticals. Nevertheless, further investigation is warranted to elucidate the metabolic pathways of bacteria to understand the precise microbial glucose metabolic metabolism.

## Figures and Tables

**Table 1:** Antioxidant activities of eleven different ratios of vitexin and iso-vitexin.

Sample	Ratio	IC_50_ (µg/mL)		FRAP (mg Trolox/g MBCE)
		DPPH	ABTS	
Vitexin/iso-vitexin	0:1	4.91 ± 0.71 ^g^	1.27 ± 0.20 ^f^	41.04 ± 0.04 ^a^
	1:0	10.22 ± 1.84 ^f^	9.55 ± 1.51 ^e^	37.81 ± 0.04 ^a^
	1:1	73.22 ± 7.31 ^a^	71.76 ± 10.13 ^a^	39.06 ± 0.03 ^a^
	1:1.5	63.64 ± 12.34 ^b^	54.67 ± 1.80 ^b^	18.36 ± 0.04 ^b^
	1:2	42.44 ± 4.45 ^d^	41.80 ± 1.56 ^c^	15.32 ± 0.05 ^bc^
	1:2.5	36.12 ± 0.18 ^de^	48.16 ± 1.96 ^bc^	12.52 ± 0.05 ^cd^
	1:3	30.19 ± 0.42 ^c^	31.24 ± 0.52 ^d^	10.38 ± 0.05 ^de^
	1.5:1	78.31 ± 2.70 ^a^	53.06 ± 1.02 ^b^	9.33 ± 0.01 ^e^
	2:1	74.14 ± 0.01 ^a^	42.10 ± 2.98 ^c^	14.64 ± 0.02 ^c^
	2.5:1	52.48 ± 6.44 ^c^	29.31 ± 12.64 ^d^	12.29 ± 0.01 ^cd^
	3:1	52.16 ± 7.30 ^c^	31.83 ± 0.88 ^d^	9.96 ± 0.04 ^e^
Trolox	-	30.00 ± 0.05 ^e^	44.00 ± 0.03 ^c^	-

All data are expressed as ± standard deviation. Within columns, means followed by different superscripts are significantly different at *p* < 0.05.

**Table 2:** The alpha-amylase inhibition activity of vitexin and iso-vitexin.

Sample	Ratio (Vitexin/Iso-Vitexin)	%Alpha-Amylase Inhibition	FIA Index
MBCE	0:1	63.07 ± 0.40 ^g^	1.00
	1:0	71.31 ± 0.22 ^a^	1.00
	1:1	63.94 ± 0.88 ^g^	1.26
	1:1.5	68.45 ± 1.30 ^fed^	2.13
	1:2	62.67 ± 0.37 ^gh^	2.43
	1:2.5	60.79 ± 0.59 ^h^	1.61
	1:3	69.10 ± 0.17 ^bcd^	2.01
	1.5:1	68.56 ± 1.84 ^fed^	2.44
	2:1	67.83 ± 0.84 ^fed^	2.41
	2.5:1	66.49 ± 0.39 ^f^	2.15
	3:1	68.50 ± 0.89 ^fed^	2.44
Commercial standard	0:1	62.53 ± 0.80 ^gh^	1.00
	1:0	70.84 ± 0.32 ^a^	1.00
	1:1	63.81 ± 1.32 ^g^	1.92
	1:1.5	68.30 ± 1.17 ^fed^	2.06
	1:2	63.06 ± 1.94 ^g^	1.88
	1:2.5	68.30 ± 1.58 ^fed^	2.06
	1:3	69.30 ± 0.40 ^bcd^	2.09
	1.5:1	68.70 ± 1.76 ^fed^	2.07
	2:1	68.23 ± 1.47 ^fed^	2.05
	2.5:1	66.75 ± 0.62 ^f^	1.93
	3:1	69.23 ± 0.66 ^bcd^	2.08
Acarbose	-	70.78 ± 0.83 ^ab^	-

All data are expressed as ± standard deviation. Within columns, means followed by different superscripts are significantly different at *p* < 0.05.

**Table 3:** The alpha-glucosidase inhibition activity of vitexin and iso-vitexin.

Sample	Ratio (Vitexin/Iso-Vitexin)	%Alpha-Glucosidase Inhibition	FIA Index
MBCE	0:1	52.30 ± 1.88 ^fg^	1.00
	1:0	60.83 ± 0.84 ^bc^	1.00
	1:1	35.45 ± 1.70 ^i^	1.91
	1:1.5	59.83 ± 1.14 ^bc^	2.05
	1:2	54.48 ± 1.14 ^def^	1.97
	1:2.5	45.29 ± 1.06 ^h^	1.82
	1:3	56.46 ± 1.43 ^ef^	2.06
	1.5:1	44.42 ± 1.49 ^h^	1.33
	2:1	32.17 ± 0.76 ^i^	0.96
	2.5:1	56.45 ± 1.68 ^ef^	2.09
	3:1	57.99 ± 1.98 ^cd^	1.73
Commercial standard	0:1	53.16 ± 1.75 ^fg^	1.00
	1:0	61.49 ± 0.84 ^bc^	1.00
	1:1	36.32 ± 0.87 ^i^	1.09
	1:1.5	63.89 ± 1.32 ^bc^	1.92
	1:2	51.20 ± 1.33 ^g^	2.13
	1:2.5	45.08 ± 1.27 ^h^	1.36
	1:3	56.89 ± 0.89 ^ef^	1.71
	1.5:1	45.51 ± 1.99 ^h^	1.37
	2:1	36.55 ± 1.54 ^i^	1.10
	2.5:1	56.89 ± 1.05 ^efg^	2.25
	3:1	56.02 ± 1.33 ^efg^	1.69
Acarbose	-	71.55 ± 1.09 ^a^	-

All data are expressed as ± standard deviation. Within columns, means followed by different superscripts are significantly different at *p* < 0.05.

**Table 4:** Percentage change at genus levels.

Genus	Control			Treatment		
	Donor 1	Donor 2	Donor 3	Donor 1	Donor 2	Donor 3
*Adlercreutzia*	−0.0332	−0.0061	−0.0052	0.0401	0.0036	0.0094
*Terrisporobacter*	−0.0302	−0.0056	−0.0690	0.04240	0.0010	0.0673
*Promicromonospora*	−0.5617	−0.2125	−1.5741	1.2150	0.3586	1.9098
*Pseudonocardia*	−0.7932	−1.5335	−1.4444	2.1024	3.2836	1.6846
*Anaerostipes*	−1.9218	−0.1260	−0.2782	3.0414	0.2375	0.2779
*Akkermansia*	−0.0042	−0.0097	−0.0197	0.0070	0.0272	0.0328
*Alistipes*	−0.0286	−0.0466	−0.8382	0.0316	0.0646	0.8999
*Parabacteroides*	−16.3417	−15.4376	−26.1212	13.5192	12.4244	23.1782
*Ruminococcus 2*	−1.4958	−14.1600	−18.5971	2.2853	10.2125	16.5730
*Roseburia*	−7.3000	−0.1716	−1.4926	5.0034	0.2746	1.4147
*Megasphaera*	−0.5343	−1.5168	−3.0740	1.0407	1.7551	3.6659
*Weissella*	−0.0064	−0.0020	−0.0249	0.0190	0.0056	0.0407
*Neglecta*	−6.7556	0.0000	−0.06885	5.9144	0.0000	0.0965
*Romboutsia*	−1.0271	−3.0153	−1.3982	1.5801	2.2438	1.1298
*Enterocloster*	−3.8984	−1.5035	−3.2172	5.3252	2.6369	3.1128
*Peptacetobacter*	0.0000	−6.5689	−0.7458	0.0000	12.1658	1.2034
*Collinsella*	−11.2280	−2.7956	−0.0431	6.8622	1.3445	0.0448
*Paraclostridium*	−0.0916	−0.2868	−0.8999	0.2199	0.7557	1.1106
*Duncaniella*	−1.7761	−0.0102	−1.2393	1.4411	0.0195	1.0158
*Streptococcus*	−0.8264	−0.1352	−0.0379	1.0430	0.1934	0.0423
*Gillisia*	−1.9951	−1.2536	−0.0099	2.1649	0.6211	1.0158
*Lawsonibacter*	6.6491	0.0000	2.1445	−6.5535	0.0000	−2.1697
*Proteus*	2.4677	17.6293	33.3981	−2.4674	−17.4997	−33.3996
*Butyricicoccus*	2.2504	1.2140	21.3012	−1.8365	−0.9277	−20.3182
*Bifidobacterium*	0.0260	0.0025	0.0000	−0.0225	−0.0015	−0.0005
*Fusobacterium*	0.4532	2.0020	2.0617	0.2838	−1.9637	−1.9650
*Flavonifractor*	0.0016	0.5628	0.0000	−0.0011	−0.5103	0.0000

## Data Availability

The data generated for this research are available on request to the corresponding author.

## Supplementary Materials

The following supporting information can be downloaded at: https://www.mdpi.com/article/10.3390/nu16173017/s1. Table S1: relative abundance at phylum levels; Table S2: relative abundance at family levels; Table S3: percentage change at genus levels.
