# Supplementary material for: Effects of Purified Vitexin and Iso-Vitexin from Mung Bean Seed Coat on Antihyperglycemic Activity and Gut Microbiota in Overweight Individuals’ Modulation
Source: Nutrients. 2024 Sep 6;16(17):3017. doi: 10.3390/nu16173017 (PMC11396884; doi:10.3390/nu16173017)
Supplement: Supplementary file 1 [file nutrients-16-03017-s001.zip › nutrients-3158335-supplementary.pdf]

Supplementary materials

Supplementary Table S1. Relative abundance at phylum levels.

Phylum	Control			Treatment		
	Donor 1	Donor 2	Donor 3	Donor 1	Donor 2	Donor 3
Firmicutes	21.3986	24.9092	50.0761	31.5851	28.2239	49.5107
Proteobacteria	78.3565	73.7341	49.1644	68.2224	70.1170	47.7291
Bacteroidetes	0.0125	0.0314	0.0104	0.0108	0.0107	0.0000
Actinobacteria	0.0177	0.7823	0.4790	0.0614	1.4547	2.3280
Verrucomicrobia	0.0000	0.0015	0.0005	0.0000	0.0000	0.0010
Fusobacteria	0.0000	0.3156	0.0047	0.0000	0.0076	0.0000
Gemmatimonadetes	0.0000	0.0000	0.0000	0.0000	0.0000	0.0000
Synergistetes	0.0000	0.0000	0.0016	0.0000	0.0000	0.0000

Supplementary Table S2. Relative abundance at family levels.

Family	Control			Treatment		
	Donor 1	Donor 2	Donor 3	Donor 1	Donor 2	Donor 3
Acidaminococcaceae	9.5747	2.4803	4.6785	19.1722	2.1647	1.0408
Clostridiaceae 1	7.3435	0.5876	3.8234	4.4148	0.0041	20.5760
Desulfovibrionaceae	0.0000	0.0000	0.0026	0.0026	0.0000	0.0025
Enterococcaceae	2.4730	17.8295	33.4064	3.7032	7.5518	26.1675
Erysipelotrichaceae	0.3198	0.0582	0.1434	1.3464	0.1973	0.1061
Eubacteriaceae	0.0000	0.0000	0.2244	0.0000	0.0000	0.0000
Lachnospiraceae	0.7014	2.8625	0.3465	1.2060	8.6140	0.6646
Lactobacillaceae	0.1731	0.1724	0.0010	0.1068	0.0540	0.0025
Peptoniphilaceae	0.0000	0.0000	0.0052	0.0021	0.0000	0.0000
Peptostreptococcaceae	0.0484	0.06432	5.8831	0.0408	9.4250	0.0652
Ruminococcaceae	0.5189	0.1133	0.3668	0.8200	0.0668	0.0096
Selenomonadaceae	0.0000	0.0096	0.0005	0.0000	0.0280	0.0000
Streptococcaceae	0.0530	0.0592	0.0208	0.1316	0.0301	0.0152
Veillonellaceae	0.0915	0.0000	0.6229	0.5460	0.0102	0.0854
Enterobacteriaceae	77.7955	72.9281	48.0886	67.1206	69.2898	46.8461
Morganellaceae	0.0031	0.2311	0.0000	0.0098	0.0836	0.0000
Pasteurellaceae	0.0395	0.0000	0.0000	0.2193	0.0000	0.0000
Phyllobacteriaceae	0.0000	0.0000	0.0000	0.0000	0.0005	0.0000
Rhodobacteraceae	0.0031	0.0091	0.0016	0.0057	0.0036	0.0000
Sphingomonadaceae	0.0000	0.0000	0.0000	0.0000	0.0000	0.0000
Sutterellaceae	0.0000	0.0000	0.0000	0.0000	0.0000	0.0000
Muribaculaceae	0.0000	0.0000	0.0000	0.0000	0.0000	0.0000
Odoribacteraceae	0.0000	0.0000	0.0000	0.0000	0.0000	0.0000
Prevotellaceae	0.0000	0.0000	0.0010	0.0010	0.0005	0.0000

Family	Control			Treatment		
	Donor 1	Donor 2	Donor 3	Donor 1	Donor 2	Donor 3
Rikenellaceae	0.0000	0.0000	0.0016	0.0000	0.0000	0.0000
Eggerthellaceae	0.0042	0.0030	0.0026	0.0191	0.0025	0.0091
Micrococcaceae	0.0000	0.0000	0.0000	0.0000	0.0000	0.0000
Nakamurellaceae	0.0000	0.0000	0.0000	0.0010	0.0056	0.0015
Porphyromonadaceae	0.0000	0.0000	0.0000	0.0010	0.0000	0.0000
Pseudonocardiaceae	0.0000	0.0000	0.0000	0.0000	0.0000	0.0000
Solirubrobacteraceae	0.0000	0.0000	0.0000	0.0000	0.0000	0.0000
Rubritaleaceae	0.0000	0.0000	0.0000	0.0000	0.0000	0.0000
Fusobacteriaceae	0.0000	0.3155	0.0047	0.0000	0.0076	0.0000
Gemmatimonadaceae	0.0000	0.0000	0.0000	0.0000	0.0000	0.0000
Synergistaceae	0.0000	0.0000	0.0016	0.0000	0.0000	0.0000

Supplementary Table S3. Percentage change at genus levels.

Genus	Control			Treatment		
	Donor 1	Donor 2	Donor 3	Donor 1	Donor 2	Donor 3
Adlercreutzia	-0.0332	-0.0061	-0.0052	0.0401	0.0036	0.0094
Terrisporobacter	-0.0302	-0.0056	-0.0690	0.0424	0.0010	0.0673
Promicromonospora	-0.5617	-0.2125	-1.5741	1.2150	0.3586	1.9098
Pseudonocardia	-0.7932	-1.5335	-1.4444	2.1024	3.2836	1.6846
Anaerostipes	-1.9218	-0.1260	-0.2782	3.0414	0.2375	0.2779
Akkermansia	-0.0042	-0.0097	-0.0197	0.0070	0.0272	0.0328
Alistipes	-0.0286	-0.0466	-0.8382	0.0316	0.0646	0.8999
Parabacteroides	-16.3417	-15.4376	-26.1212	13.5192	12.4244	23.1782
Ruminococcus 2	-1.4958	-14.1600	-18.5971	2.2853	10.2125	16.5730
Roseburia	-7.3000	-0.1716	-1.4926	5.0034	0.2746	1.4147
Megasphaera	-0.5343	-1.5168	-3.0740	1.0407	1.7551	3.6659
Weissella	-0.0064	-0.0020	-0.0249	0.0190	0.0056	0.0407
Neglecta	-6.7556	0.0000	-0.06885	5.9144	0.0000	0.0965
Romboutsia	-1.0271	-3.0153	-1.3982	1.5801	2.2438	1.1298
Enterocloster	-3.8984	-1.5035	-3.2172	5.3252	2.6369	3.1128
Peptacetobacter	0.0000	-6.5689	-0.7458	0.0000	12.1658	1.2034
Collinsella	-11.2280	-2.7956	-0.0431	6.8622	1.3445	0.0448
Paraclostridium	-0.0916	-0.2868	-0.8999	0.2199	0.7557	1.1106
Duncaniella	-1.7761	-0.0102	-1.2393	1.4411	0.0195	1.0158
Streptococcus	-0.8264	-0.1352	-0.0379	1.0430	0.1934	0.0423
Gillisia	-1.9951	-1.2536	-0.0099	2.1649	0.6211	1.0158
Lawsonibacter	6.6491	0.0000	2.1445	-6.5535	0.0000	-2.1697
Proteus	2.4677	17.6293	33.3981	-2.4674	-17.4997	-33.3996
Butyricicoccus	2.2504	1.2140	21.3012	-1.8365	-0.9277	-20.3182

Genus	Control			Treatment		
	Donor 1	Donor 2	Donor 3	Donor 1	Donor 2	Donor 3
Bifidobacterium	0.0260	0.0025	0.0000	-0.0225	-0.0015	-0.0005
Fusobacterium	0.4532	2.0020	2.0617	0.2838	-1.9637	-1.9650
Flavonifractor	0.0016	0.5628	0.0000	-0.0011	-0.5103	0.0000
Lachnospira	0.0000	-0.0307	-0.2258	0.0000	0.0159	0.1893
Tyzzerella	0.0000	-0.0031	-0.0862	0.0000	0.0056	0.0751
Holdemania	0.0000	0.0000	0.1263	0.0000	0.0000	-0.1285
Prevotella	0.0000	0.0000	-0.0114	0.0000	0.0005	0.0057
Mesorhizobium	0.0000	0.0000	-0.0078	0.0000	0.0000	0.0177
Phocea	0.0000	0.0000	-0.0182	0.0000	0.0005	0.0302
Allisonella	0.0005	-22.2866	-0.5276	-0.0005	18.3060	0.5421
Acidaminococcus	-0.0087	-0.0169	-0.3078	0.0100	0.0210	0.3603
Coprococcus	-0.0001	0.0000	0.0000	-0.0005	0.0000	0.0000
Gemella	-0.0432	0.0000	0.0000	0.0901	0.0000	0.0000
Longibaculum	0.0000	-0.0118	-0.0145	0.0000	0.0031	0.0167
Rothia	0.0000	-5.7517	-2.1885	0.0000	6.4206	2.8079
Sulfitobacter	0.0000	0.0471	0.0000	0.0000	-0.0455	0.0000
Lactiplantibacillus	0.0000	0.0000	-0.0109	0.0000	0.0000	0.0224
Bilophila	0.0000	-0.0118	-0.0311	0.0000	0.0087	0.0235
Rubritalea	3.9880	0.5789	3.2816	-3.7752	-0.5727	-3.2968
Bacteroides	-0.0017	0.0000	-0.0353	0.0015	0.0000	0.0746
Faecalibacterium	0.0000	-0.0015	-0.3130	0.0000	0.0000	0.4395
Blautia	-0.0064	0.0000	0.0000	0.0045	0.0000	0.0000
Parasutterella	-0.0017	0.0000	-0.0016	0.0015	0.0005	0.0010
Sphingobium	0.0000	0.0000	-0.0628	0.0000	0.0000	0.0464
Turicibacter	0.0000	-0.0046	-0.0477	0.0005	0.0051	0.0542
Agathobacter	-0.0234	-0.0082	-0.0156	0.0726	0.0046	0.0162
Catenibacterium	0.0000	0.0000	-0.0036	0.0000	0.0000	0.0021
Intestinimonas	-0.0006	-0.0031	0.0587	0.0000	0.0041	-0.0508
Sutterella	0.0000	0.0000	-0.4565	0.0000	0.0000	0.7792
Nakamurella	-0.0834	0.0000	-0.0389	0.0811	0.0000	0.0323
Eggerthella	-0.0006	0.0000	0.0052	0.0000	0.0000	-0.0052
Odoribacter	-0.0017	0.0000	-0.0358	0.0050	0.0005	0.0334
Odoribacter	0.0000	-0.0092	-0.0462	0.0000	0.0041	0.0615
Dialister	0-0.8265	-0.4224	-1.4672	0.9225	0.6484	1.7373
Enterococcus	0.0000	0.0504	-0.4240	0.0000	-0.0463	0.5417
Phascolarctobacterium	-0.0111	-0.0026	-0.0031	0.0140	0.0031	0.0068
Plesiomonas	-2.6932	-1.3939	-0.2154	3.2903	1.1242	0.2091
Roseisolibacter	0.0056	0.0075	-0.0026	0.0018	-0.0116	0.0000
Dysosmobacter	-0.3448	-0.0118	0.0000	0.3611	0.0169	0.0005

Genus	Control			Treatment		
	Donor 1	Donor 2	Donor 3	Donor 1	Donor 2	Donor 3
Paraprevotella	0.0000	-2.2520	-6.1622	0.0000	2.8584	6.4513
Solirubrobacter	0.0000	-0.0005	-0.0130	0.0000	-0.0005	0.0099
Faecalibacillus	0.0000	-0.1301	-0.0903	0.0000	0.2411	0.1330
Dielma	0.0000	0.0000	-0.0031	0.0000	0.0000	0.0016
Morganella	-0.7788	-0.0246	-0.2979	0.7933	0.0380	0.3003
Veillonella	-0.6101	-0.1761	-0.4411	0.3576	0.1518	0.5657
Oscillibacter	-0.2183	0.0000	-0.0047	0.3368	0.0000	0.0042
Escherichia/Shigella	-0.0012	0.0000	-0.0016	0.0010	0.0000	0.0010
Anaerotruncus	0.0320	-0.0719	-0.0343	-0.0238	0.1024	0.0391
Flintibacter	-0.0006	0.0000	-0.0078	0.0000	0.0005	0.0162
Eisenbergiella	0.0000	-0.0015	-0.0042	0.0000	0.0010	0.0026
Senegalimassilia	0.0000	0.0000	-0.0031	0.0000	0.0000	0.0052
Clostridium XVIII	0.0000	0.0000	-0.0052	0.0000	0.0000	0.0052
Sediminicola	-0.0017	0.0000	-0.8745	0.0145	0.0000	1.0245
Lacticaseibacillus	0.0000	0.0000	0.0000	0.0085	0.0000	0.0000
Anaerofilum	0.0000	0.1451	0.0000	0.0000	-0.1431	0.0000
Schaalia	0.0000	-0.0010	-0.0016	0.0005	0.0000	0.0000
Lachnospiracea incertae sedis	0.0000	-0.0031	-1.0255	0.0000	0.0036	0.9057
Burkholderia	0.0000	-0.0154	-0.5097	0.0000	0.0185	0.6846
Clostridium IV	0.0000	0.0000	-0.0042	0.0000	0.0000	0.0047
Lactonifactor	0.0000	0.0000	-0.0119	0.0000	0.0000	0.0182
Gemmiger	-0.0087	-0.0246	-0.6161	0.0170	0.0339	0.5673
Haemophilus	0.0174	-0.0056	5.2598	-0.0178	0.0077	-5.2592
Phocaeicola	0.0000	0.0000	-0.0602	0.0000	0.0000	0.0454
Eubacterium	0.0000	-0.0184	-0.0036	0.0000	0.0154	0.0115
Amedibacillus	-0.0001	0.0000	0.0000	0.0165	0.0000	0.0000
Finegoldia	-0.5827	-0.0502	-0.1474	0.5148	0.0308	0.0673
Clostridium sensu stricto	0.0000	0.0480	0.3138	0.0000	-0.0475	-0.3230
Megamonas	1.3008	0.0005	0.0005	-1.3015	-0.0005	-0.0005
Ruminococcus	0.0000	0.0000	0.0000	0.0090	0.0000	0.0000
Sellimonas	0.0031	0.0051	0.0000	-0.0031	-0.0051	0.0000
Winogradskyella	0.0000	0.0687	0.0000	0.0000	-0.0698	0.0000
Massiliimalia	0.0000	0.0000	0.0000	0.0611	0.0000	0.0000
Providencia	0.0000	0.0000	-0.0254	0.0000	0.0000	0.0261
Intestinibacter	-0.0140	-0.0041	0.0000	0.0135	0.0000	0.0000
Anaerotignum	-0.0087	0.0000	0.0000	0.0000	0.0000	0.0000
Raoultibacter	0.0000	0.0000	-2.9624	0.0000	0.0000	3.4849
Anaerobutyricum	-0.0023	-0.0010	-0.2242	0.0000	0.0000	0.2398

Genus	Control			Treatment		
	**Donor 1**	**Donor 2**	**Donor 3**	**Donor 1**	**Donor 2**	**Donor 3**
Cloacibacillus	-0.0321	-0.0077	-0.0083	0.0761	0.0077	0.0141
Dorea	-0.0006	-0.0016	0.0000	0.0015	-0.0015	0.0000
Ruthenibacterium	-0.0052	0.0000	0.0000	0.0100	0.0000	0.0000
Hungatella	0.0000	-0.2628	-0.2694	0.0000	0.2151	0.2884
Lactococcus	0.0000	-0.1470	0.0000	0.0000	0.1211	0.0000
Mediterraneibacter	-0.8749	-0.6175	-1.3193	1.0552	0.8454	1.0652
Fusicatenibacter	0.0000	0.0000	0.0000	0.0300	0.0000	0.0000
Pediococcus	0.0000	0.0000	0.0000	0.0336	0.0000	0.0000
Actinomyces	0.0000	0.0000	0.0000	0.0125	0.0000	0.0000
Negativibacillus	0.0000	-0.0020	-0.0062	0.0000	0.0015	0.0021
Desulfovibrio	0.0000	-0.1341	-0.0109	0.0000	0.0898	0.0099
Erysipelatoclostridium	-0.0010	0.0045	0.0005	0.0064	-0.0070	-0.0016
Citrobacter	0.0000	-0.1070	-0.0042	0.0005	0.0539	0.0052
Slackia	-0.0105	0.0000	-1.0161	0.0050	0.0000	1.1035
Faecalimonas	0.0198	-0.0072	0.4068	0.0207	0.0021	-0.4068
Butyricimonas	0.0010	0.0025	-0.0010	-0.0011	-0.0030	0.0000
